# Novel left ventricular mechanical index in pulmonary arterial hypertension

**DOI:** 10.1002/pul2.12216

**Published:** 2023-04-01

**Authors:** Kenzo Ichimura, Everton J. Santana, Tatiana Kuznetsova, Nicholas Cauwenberghs, František Sabovčik, Lindsey Chun, Nadia L. C. Francisco, Vitaly O. Kheyfets, Michael Salerno, Roham T. Zamanian, Edda Spiekerkoetter, Francois Haddad

**Affiliations:** ^1^ Department of Medicine, Division of Pulmonary, Allergy and Critical Care Stanford University Stanford California USA; ^2^ Vera Moulton Wall Center of Pulmonary Vascular Disease Stanford School of Medicine Stanford California USA; ^3^ Cardiovascular Institute Stanford University Stanford California USA; ^4^ Department of Medicine, Division of Cardiovascular Medicine Stanford University Stanford California USA; ^5^ Research Unit Hypertension and Cardiovascular Epidemiology, Department of Cardiovascular Sciences University of Leuven Leuven Belgium; ^6^ Pediatric Critical Care Medicine; Developmental Lung Biology and CVP Research Laboratories, School of Medicine University of Colorado Aurora Colorado USA

**Keywords:** echocardiography, pulmonary hypertension, ventricular function

## Abstract

Ventricular interdependence plays an important role in pulmonary arterial hypertension (PAH). It can decrease left ventricular (LV) longitudinal strain (LVLS) and lead to a leftward displacement (“transverse shortening”) of the interventricular septum (sTS). For this study, we hypothesized the ratio of LVLS/sTS would be a sensitive marker of systolic ventricular interactions in PAH. In a cross‐sectional cohort of patients with PAH (*n* = 57) and matched controls (*n* = 57), we quantified LVLS and septal TS in the amplitude and time domain. We then characterized LV phenotypes using upset plots, ventricular interactions using network analysis, and longitudinal analysis in a representative cohort of 45 patients. We also measured LV metrics in mice subjected to pulmonary arterial banding (PAB) using a 7 T magnetic resonance imaging at baseline, Week 1, and Week 7 post‐PAB (*N* = 9). Patients with PAH had significantly reduced absolute LVLS (15.4 ± 3.4 vs. 20.1 ± 2.3%, *p* < 0.0001), higher sTS (53.0 ± 12.2 vs. 28.0 ± 6.2%, *p* < 0.0001) and lower LVLS/sTS (0.30 ± 0.09 vs. 0.75 ± 0.16, *p* < 0.0001) compared to controls. Reduced LVLS/sTS was observed in 89.5% of patients, while diastolic dysfunction, impaired LVLS (<16%), and LV atrophy were observed in 73.7%, 52.6%, and 15.8%, respectively. In the longitudinal cohort, changes in LVLS/sTS were closely associated with changes in N‐terminal pro B‐type natriuretic peptide (*r* = 0.73, *p* < 0.0001) as well as survival. Mice subjected to PAB showed significant RV systolic dysfunction and decreased LVLS/sTS compared to sham animals. We conclude that in PAH, LVLV/sTS is a simple ratio that can reflect ventricular systolic interactions.

## INTRODUCTION

Pulmonary arterial hypertension (PAH) affects the pulmonary vasculature and often leads to right heart failure and premature death.^1^ Although the origin of the disease lies in the pulmonary vasculature, the prognosis of patients with PAH is mainly driven by their right ventricular (RV) dysfunction and failure.^2^ This has been shown using several indices including tricuspid annular plane systolic excursion (TAPSE),^3^ RV lateral free wall longitudinal strain (RVLS),^4^ RV end‐systolic volume or RV remodeling index.^5^, ^6^ In contrast to RV systolic function, left ventricular ejection fraction (LVEF) is often described as normal or hyperdynamic in patients with PAH. While LVEF may be preserved until end‐stage PAH,^7^ LVEF does not reflect the mechanical properties of the LV, which are adversely affected by PAH due to ventricular interdependence.^8^

With the development of ventricular strain imaging, there is a growing interest to assess subclinical ventricular dysfunction in PAH.^9^ Using echocardiography, Puwanant et al.^10^ have shown that impairment in LV longitudinal strain (LVLS) is common in PAH and associated with RV pressure overload. Mechanical changes in PAH are, however, not limited to the longitudinal motion. In fact, because of the prolonged contraction of the RV in PAH, septal transverse displacement is accentuated.^11^ Moreover, the severity of the septal shift has been shown as a prognostic factor for patients with RV pressure overload.^12^ Several parameters, such as the eccentricity index^13^ and the radius of the septal curvature,^14^ have been used previously as a measure of septal flattening and RV pressure overload.

**Figure 1:**
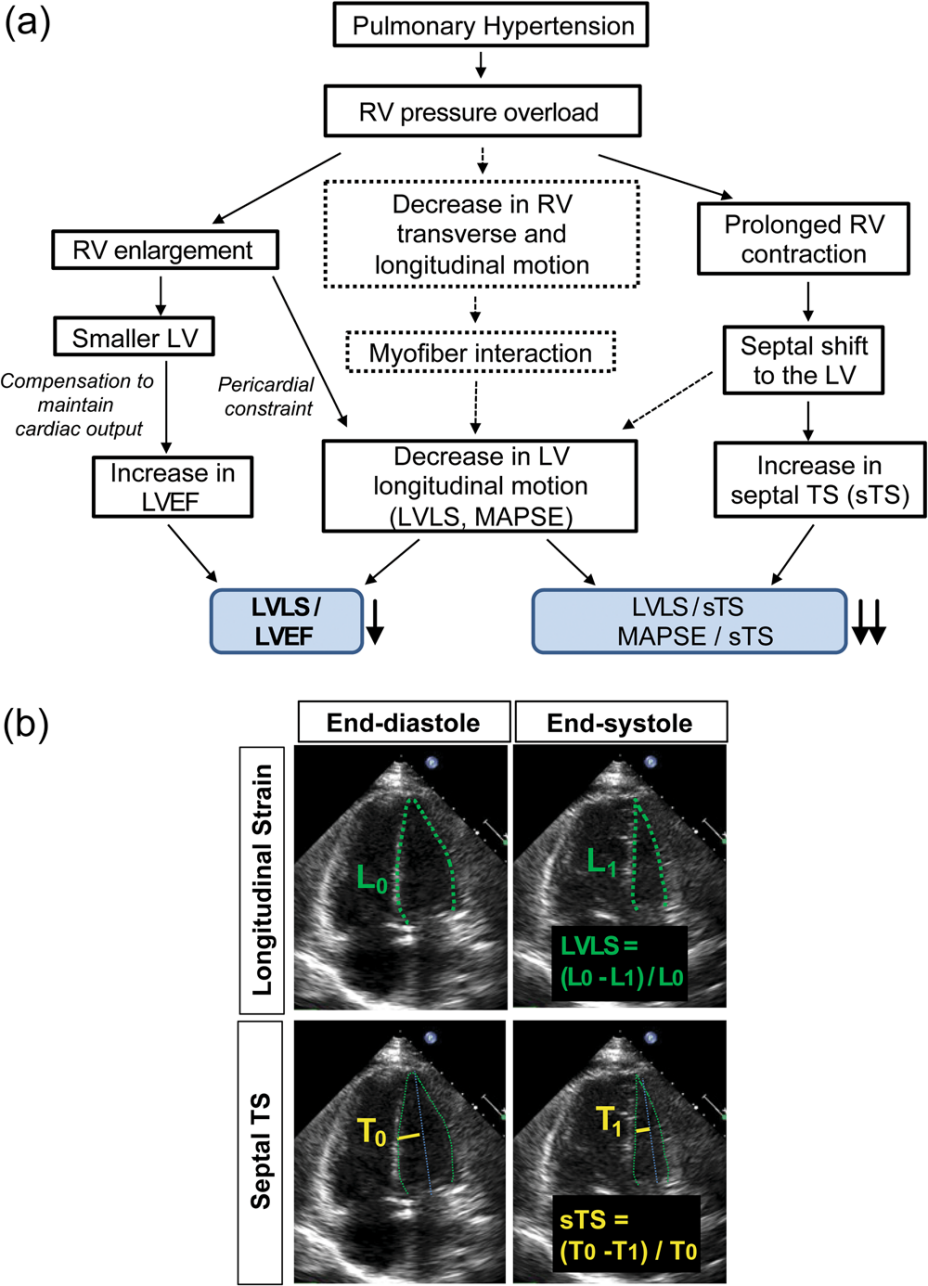
Hypothesis behind the ratios tested in this study and manual tracing of left ventricular longitudinal strain (LVLS) and septal transverse shortening (sTS). (a) In pulmonary hypertension, structural and functional interdependence influence both ventricles. As the right ventricular (RV) dilates, LV becomes smaller and by compensation left ventricular ejection fraction (LVEF) increases. On the other hand, a decrease in RV transverse and longitudinal motion will affect the LV by fiber interaction. At the same time, prolonged RV contraction leads to a septal shift to the LV and further decreases the LV longitudinal motion while increasing the sTS. In the end, this will make the ratio shown in the figure (LVLS/LVEF, LVLS/sTS, mitral annular plane systolic excursion [MAPSE]/sTS) more sensitive than all other single parameters mentioned above. Dotted lines indicate the hypothetical connections and mechanisms. (b) LVLS was calculated as 100 × (*L*_0_ − *L*_1_)/*L*_0_ by manually tracing the endocardial borders in end‐diastole and end‐systole from the septal to the lateral mitral annulus. sTS was calculated as 100 × (*T*_0_ − *T*_1_)/*T*_0_, which was measured as the fractional change of the transverse shortening of the septal wall using a centerline method centered to the mid of the annular plane and the apex.

Because LV longitudinal motion is impaired while transverse septal motion is accentuated in PAH, we hypothesized that the ratio of LVLS to septal transverse “shortening” or displacement (sTS) could be a sensitive marker of mechanical changes in PAH (Figure 1a). The first objective of our study was to describe changes in LV mechanical function in PAH focusing on both longitudinal and transverse motion. Our second objective was to better characterize LV phenotypes in PAH based on LVLS/sTS, epidemiologically defined diastolic function and LV mass. Our third objective was to determine to which extent serial changes in LVLS/sTS were related to changes in N‐terminal pro B‐type natriuretic peptide (NT‐proBNP) and TAPSE. Finally, we explored whether similar LV changes could be recapitulated in a small animal model of RV pressure overload (i.e., pulmonary artery banding [PAB]).

## METHODS

### Study population

This study was approved by the Stanford University Institutional Review Board (IRB #14083, #20942) and all patients gave written informed consent. Patient cohorts from Stanford Adult Pulmonary Hypertension Program and Vera Moulton Wall Center for Pulmonary Vascular Disease DataBase^6^ were analyzed retrospectively in this study. The cross‐sectional cohort included 57 patients with PAH evaluated at Stanford University with transthoracic echocardiography and invasive right heart catheter (RHC) performed within 24 h. We used 1:1 age‐ and sex‐matched healthy subjects derived from community volunteers as health controls for this cohort. The longitudinal cohort included 45 patients with PAH whose NT‐ProBNP levels were available at both baseline and follow‐up. In the second cohort, RHC was performed within 3 months of the first echocardiogram (77% of patients were within 1 week). Inclusion criteria of both cohorts were diagnosis of PAH according to the guidelines (mean pulmonary arterial pressure ≥ 25 mmHg and pulmonary arterial wedge pressure ≤ 15 mmHg measured by RHC) and by ruling out other causes of pulmonary hypertension. Echocardiographic images were acquired using Hewlett Packard Sonos 5500 or Philips IE33 ultrasound systems. All manual measurements were done with TomTec software (TomTec Imaging System). All conventional LV and RV parameters were averaged over three cycles and analyzed according to the latest guidelines.^15^ Measurements were performed by two blinded certified readers (K. I. and F. H.) one focusing on left heart measures and the other reader on right heart measures to minimize information bias.

### Measurement of LVLS and sTS

To maximize quality, LVLS was measured manually according to the definition of Lagrangian strain, which has been previously reported as comparable to software‐measured LV global LS.^16^, ^17^ In brief, the endocardial borders in end‐diastole and end‐systole were traced from the septal to lateral mitral annulus, excluding trabeculations and the papillary muscles. Initial length (*L*_0_) was measured in end‐diastole and final length (*L*_1_) in end‐systole. LVLS was calculated as 100 × (*L*_0_− *L*_1_)/*L*_0_ (Figure 1b). sTS was measured as the fractional change of the transverse shortening of the septal wall using a centerline method centered to the mid of the annular plane and the apex (Figure 1b). Lateral mitral annular plane systolic excursion (MAPSE) was measured using B‐mode of the apical four‐chamber view and by marking the lowest (end‐diastole) and highest (end‐systole) point of the lateral mitral annulus.

### Regional analysis of the LV by feature tracking

We used the TomTec semiautomated feature tracking tool 2D Cardiac Performance Analysis (CPA) to measure regional LS and TS. CPA generates time‐series curves of regional LS and transverse displacement, as well as LV volume as detailed in the Supporting Information: Methods. Although CPA also generated LVLS curves and peak LVLS was available, the semiautomated feature tracking was considered suboptimal in seven cases in controls and nine cases in patients with PAH due to suboptimal tracking. In cases where tracking was considered of good quality for analysis, we found a consistent correlation between manually traced Lagrangian strain and LVLS measured with semiautomated feature tracking (Supporting Information: Figure S1).

### LV phenotypes in PAH

In addition to LV longitudinal and transverse function, we also assessed LV diastolic dysfunction and LV mass. LVLS below 16% in absolute value was defined as abnormal based on the AHA/ACC/HFSA 2022 guideline of heart failure.^18^ Diastolic dysfunction was determined if *E*/*A* or *e'* was abnormal according to previously published epidemiologically based thresholds^19^ (Supporting Information: Table S1) as following ASE guidelines for diastolic dysfunction is not recommended for patients with PAH. LV atrophy was defined as 2.5th percentile of the normal value of LV mass index previously reported.^20^ Since the threshold of low LVLS/sTS, MAPSE, and MAPSE/sTS in PAH have not been previously well described, we used the 2.5th percentile of the control cases. We used UpSet plots to summarize the LV phenotypes of patients with PAH in the cross‐sectional cohort. An UpSet plot is an alternative visualization of a Venn diagram that emphasizes the cardinalities of intersections among groups.^21^

### Longitudinal cohort

Patients from the longitudinal cohort were grouped into three categories based on the longitudinal change of their NT‐proBNP. Patients with their NT‐proBNP decreased by more than 30%, changed by less than 30% in either direction, and increased by more than 30% were categorized as improved, stable, and worsened, respectively.^22^

### Animal model of PAB

All animal experiments were performed in accordance with the Guide for Care and Use of Laboratory Animals (National Research Council) and approved by the Administrative Panel on Laboratory Animal Care at Stanford University (Protocol #27626). C57BL6/J mice (JAX 000664) were purchased from the Jackson Laboratory. Male C57ML6/J mice (8–10 weeks of age, *N* = 9) underwent surgery of PAB around a 25‐G needle as previously described.^23^ Briefly, animals were anesthetized with isoflurane (induction 5%, maintenance 2%–3%) and a partial thoracotomy was performed at the second intercostal space. The main pulmonary artery trunk was isolated and a manual constriction using a 6‐0 nylon silk blade around a 25‐G needle was created. Animals were closely monitored until they recovered from surgery.

### Cardiac magnetic resonance imaging (MRI) of mice

Cardiac MRI in mice was performed with a 7 T magnetic resonance system (Bruker) in a supine position under continuous isoflurane anesthesia (1%–2%). The dose of isoflurane was adjusted to target the respiratory rate of 50–60 breaths/min. All measurements were done using Horos software (https://horosproject.org). LVEF and right ventricular ejection fraction (RVEF) were measured using the modified Simpson's method. LVLS and TS were measured as described above.

### Statistical analysis

Statistical analyses were performed using Python 3.10 and GraphPad Prism 8 software. Descriptive statistics for continuous variables were summarized as mean ± SD if they followed a normal distribution, otherwise data were presented as median and interquartile range. Categorical variables were summarized as a proportional number of subjects (%). For the comparison of unpaired two groups, unpaired two‐tailed Student's *t*‐test or Mann–Whitney *U*‐test was used for continuous data and *χ* ^2^ test for categorical data. For the comparison of three unpaired groups, one‐way analysis of variance with Tukey's multiple comparisons or Kruskal–Wallis test with Dunn's multiple comparisons were used. In the longitudinal analysis of paired samples, Wilcoxon's matched‐pair signed‐rank test was used to compare two matched groups, and Friedman's test with Dunn's multiple comparisons was used to compare three groups.

Pairwise relationships between variables were summarized using heatmaps and network correlation graphs. The correlations were calculated with the corr method of pandas (version 1.4.3).^24^ A correlation network was constructed to visualize pairwise associations between hemodynamics, RV, LV, and other variables with Spearman's correlation coefficients as the edges' weight (NetworkX version 2.8.5).^25^ An exploratory survival analysis using Cox's proportional hazard model was used for the combined end‐point of death or lung transplantation at 5 years.

## RESULTS

### LV mechanical shortening or displacement indices in patients with PAH

The baseline characteristics are summarized in Table 1. Table 2 shows the comparison between the healthy controls and the cross‐sectional PAH cohort. The PAH cohort had a mean pulmonary arterial pressure of 51.9 ± 12.7 mmHg, q pulmonary vascular resistance index of 22.2 ± 9.3 Woods unit (WU) m^2^, and a cardiac index of 2.01 [1.8–2.4, 95% confidence interval] mL/m^2^.

**Table 1:** Baseline characteristics of patients with PAH.

	Cross‐sectional Cohort (*N* = 57)	Longitudinal Cohort (*N* = 45)
Age	46.5 ± 11.2	52.6 ± 12.6
Female sex	70.1%	85.1%
PAH etiology		
Idiopathic	7 (12.3%)	8 (18%)
Drugs and toxins	20 (35.1%)	13 (28.9%)
Connective tissue disease	14 (24.6%)	20 (44.4%)
Congenital heart disease	6 (10.5%)	4 (8.9%)
Others	10 (17.5%)	0 (0%)
Hemodynamics		
Systolic blood pressure (mmHg)	109 [102–122]	111 [108–126]
HR (bpm)	80.4 ± 16.5	84.2 ± 16.1
RAP (mmHg)	8.0 [5.0–14]	10.1 ± 6.0
mPAP (mmHg)	51.9 ± 12.7	53.0 [45.0–60.0]
PCWP (mmHg)	8.7 ± 3.3	10.6 ± 5.2
PVRI (WU/m^2^)	22.2 ± 9.3	25.9 ± 10.7
CI (L/min/m^2^)	2.01 [1.8–2.4]	1.69 [1.48–2.05]
SVRI (WU/m^2^)	35.7 ± 10.2	48.6 ± 13.8

Abbreviations: HR, heart rate; mPAP, mean pulmonary artery pressure; PCWP, pulmonary capillary wedge pressure; PAH, pulmonary arterial hypertension; PVRI, pulmonary vascular resistance index; RAP, right atrial pressure; SVRI, systemic vascular resistance index; WI, Woods unit,

**Table 2:** Comparative echocardiograms of age‐ and sex‐matched controls and patients with PAH with RHC

	Control (*N* = 57)	Cross‐sectional Cohort (*N* = 57)	*p* Value
Age	45.6 ± 11.3	46.5 ± 11.2	0.67
Female sex	70.1%	70.1%	‐
BMI (kg/m^2^)	24.3 [21.2–27.0]	28.7 [23.0–32.8]	0.0003
LVEF (%)	60.6 ± 4.1	67.8 ± 7.8	<0.0001
LVSV (mL)	61.5 [51.1–70.7]	50.3 [36.3–58.4]	<0.0001
LVLS (%)	20.1 ± 2.3	15.4 ± 3.4	<0.0001
MAPSE (mm)	12.5 ± 2.3	9.0 ± 2.9	<0.0001
Septal TS (%)	28.0 ± 6.2	53.0 ± 12.2	<0.0001
Lateral TS (%)	37.9 ± 7.1	38.3 ± 12.9	0.6233
*E*/*A*	1.3 [1.1–1.6]	0.85 [0.69–1.2]	<0.0001
*E*/e′	7.6 [6.4–8.9]	7.3 [4.9–11.3]	0.6966
LAtEF (%)	50.9 ± 13.6	36.8 ± 14.2	<0.0001
RVEDA (mm^2^)	19.7 [16.4–22.9]	36.6 [30.6–44.6]	<0.0001
RVFAC (%)	42.3 [38.8–44.3]	20.0 [16.0–26.4]	<0.0001
RVLS (%)	25.9 [24.5–29.4]	14.1 [10.7–16.7]	<0.0001

Abbreviations: BMI, body mass index; LAtEF, left atrial total emptying fraction; LVEF, left ventricular ejection fraction; LVLS, left ventricular longitudinal strain; LVSV, left ventricular stroke volume; MAPSE, mitral annular plane systolic excursion; RVEDA, right ventricular end‐diastolic area; RVFAC, right ventricular fractional area change; RVLS, right ventricular global longitudinal strain; TS, transverse shortening.

**Figure 2:**
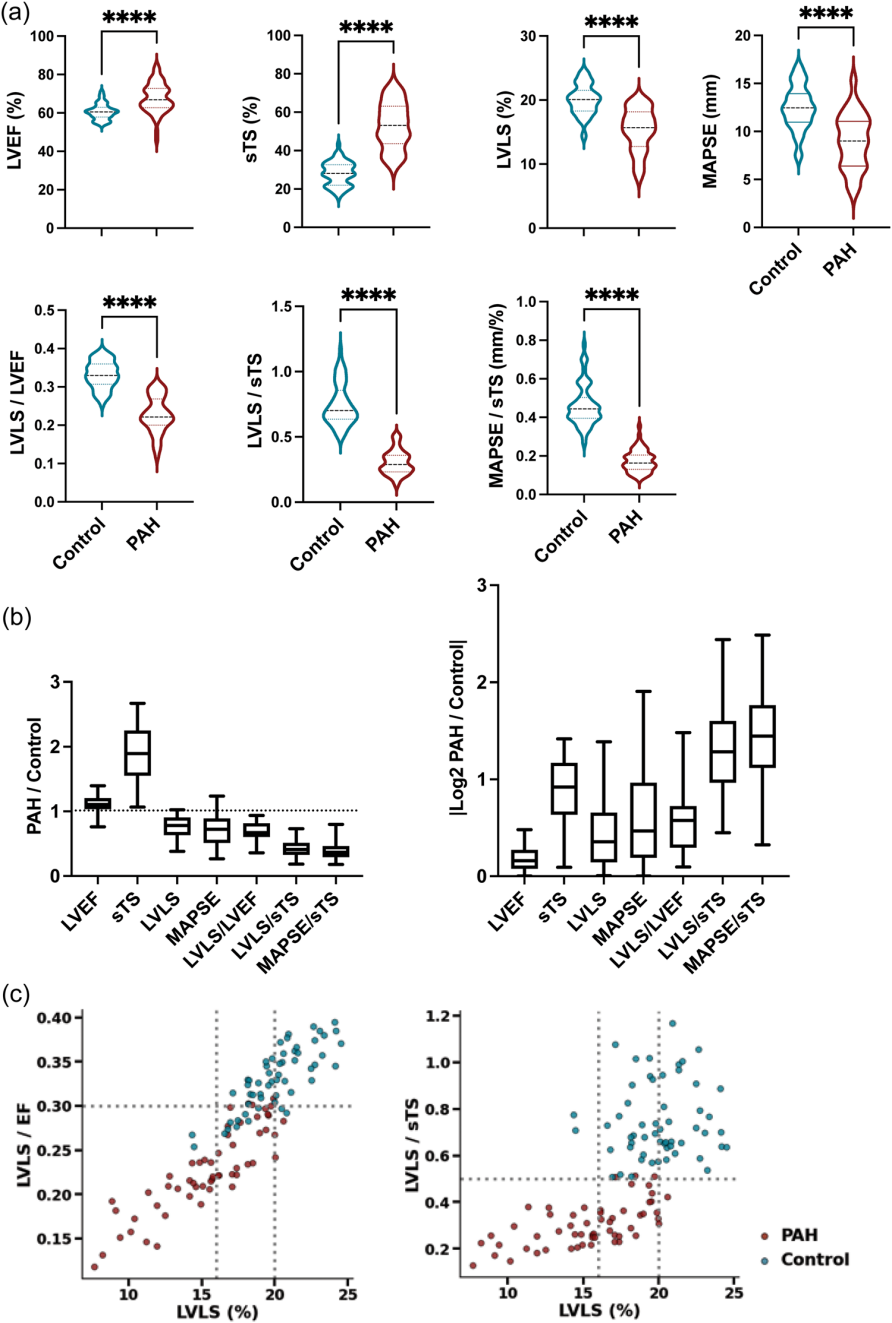
Comparison of parameters measured to capture the mechanical changes in the left ventricular (LV). (a) left ventricular ejection fraction (LVEF) and septal transverse shortening (sTS) were significantly higher in patients with pulmonary arterial hypertension (PAH), while left ventricular longitudinal strain (LVLS) and mitral annular plane systolic excursion (MAPSE) were decreased. Consequently, the ratios of these parameters were all significantly lower in patients with PAH. *N* = 57 for both groups. Data were analyzed by unpaired two‐tailed Mann–Whitney *U*‐test. **** *p* < 0.00005. (b) Comparison of the fold‐change of the parameters and ratios. The left panel shows the absolute fold change and the right panel shows the absolute value of the log 2 fold change. (c) LVLS/sTS better differentiate cases from controls than LVLS, especially in patients with LVLS between 16% and 20%.

Compared to age‐ and‐sex‐matched controls, patients with PAH had significantly higher LVEF (67.8 ± 7.8 vs. 60.6 ± 4.1%, *p* < 0.0001), sTS (53.0 ± 12.2 vs. 28.0 ± 6.2, *p* < 0.0001), and reduced absolute LVLS (15.4 ± 3.4 vs. 20.1 ± 2.3%, *p* < 0.0001) and MAPSE (9.0 ± 2.9 vs. 12.5 ± 2.3 mm, *p* < 0.0001) (Figure 2a). Accordingly, the ratios of LVLS/LVEF, LVLS/sTS, and MAPSE/sTS were all reduced in patients with PAH. When comparing the metrics, the ratios of LVLS/sTS and MAPSE/sTS also showed the largest fold change in PAH when referenced to the median value of controls (Figure 2b). All of these ratios correlated moderately with the hemodynamic parameters (Supporting Information: Figure S2). Figure 2c shows the relationship between LVLS and LVLS/sTS ratio with color overlay of cases versus controls. The discrimination between cases and controls was better with the LVLS/sTS ratio, especially in patients with LVLS between 16% and 20%.

### Feature tracking and time domain analysis

**Figure 3:**
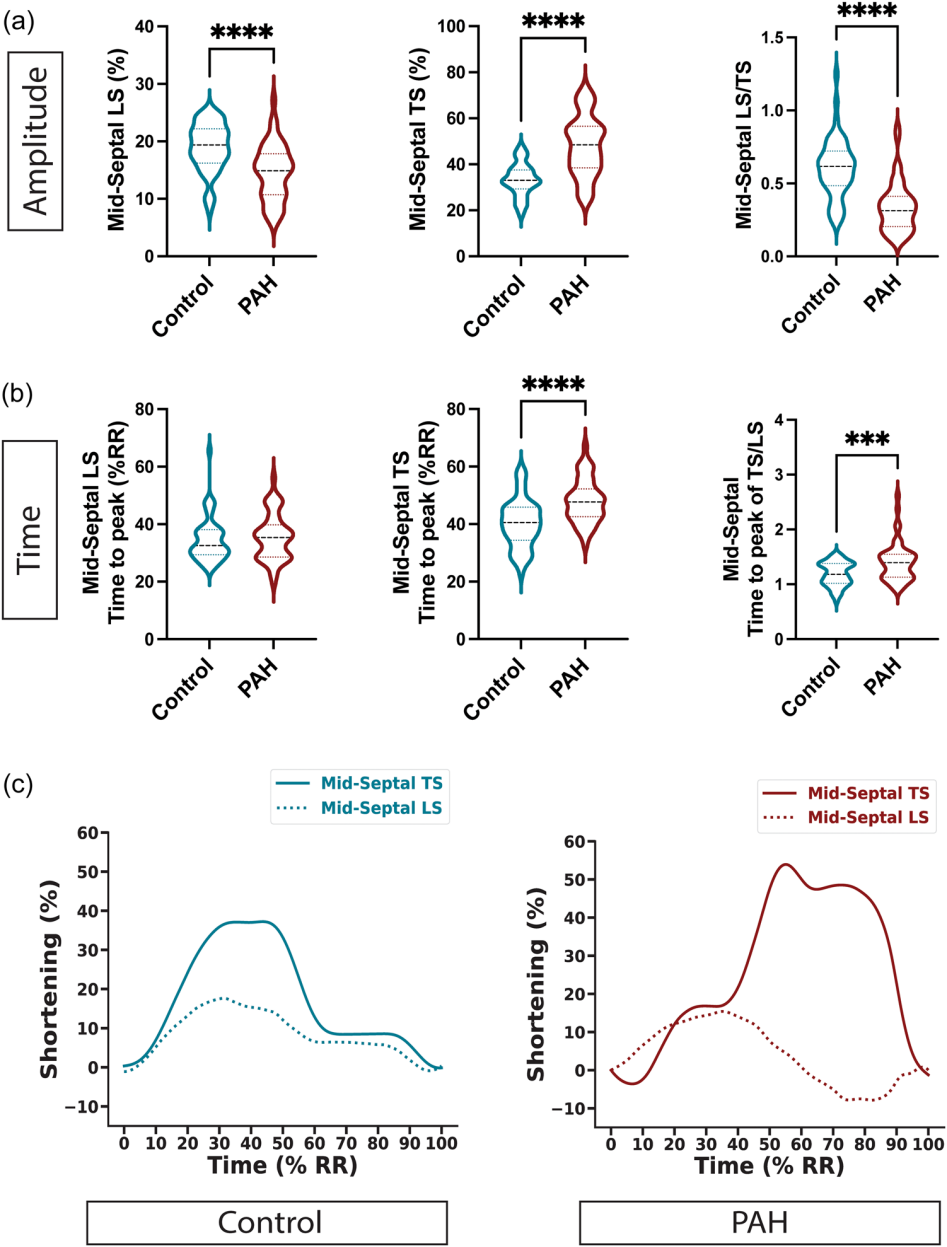
Regional displacement, strain, and time dispersion analysis of the left ventricular (LV) mid‐septum by automated feature tracking. LV shortening was assessed in both amplitude (a) and time domain (b). (a) Minor decrease in longitudinal shortening (LS) accompanied by a large increase in transverse shortening (TS) lead to a significant decrease in their ratio. (b) Time to peak of LS or TS showing the time dispersion between LS and TS. Data were analyzed by unpaired two‐tailed Mann–Whitney *U*‐test. ***p < 0.0005; ****p < 0.00005. (c) Representative curves of LS and TS of mid‐septal segments show clear time dispersion of the peak and indicate transverse displacement of the septum.

We performed regional LS and TS analysis and focused on the mid‐septal and mid‐lateral segments of the LV based on four‐chamber images. Mid‐septal LS was lower in the PAH group compared to control cases (14.6 ± 4.9% vs. 19.0 ± 4.2%, *p* < 0.0001), whereas mid‐septal TS was significantly higher (48.0 ± 13.0% vs. 33.3 ± 7.1%, *p* < 0.0001; Figure 3a). Mid‐septal LS also had a trend for greater impairment than mid‐lateral LS (14.6 ± 4.9% vs. 16.6 ± 4.7%, *p* = 0.051). Mid‐lateral TS between PAH and control cases were not significantly different (28.9 ± 11.9% vs. 25.2 ± 8.4%, *p* = 0.177; Supporting Information: Figure S3A), highlighting that the increased transverse displacement was mainly confined to the mid‐septal wall segment.

Feature tracking also allows the analysis of the timing of segmental shortening. Here timing is normalized to the RR interval. There was a significant delay in mid‐septal TS in PAH cases compared to control (48.0 ± 7.7% RR vs. 40.2 ± 8.4%RR, *p* < 0.0001), whereas no delay was observed in mid‐septal LS (34.5 ± 8.1%RR vs. 34.5 ± 7.6% RR; Figure 3b). The septal LS and TS curves of the representative cases (i.e., selected for representative median peak LS and TS values) showed substantial time dispersion between mid‐septal TS relative to LS, which was not observed either in control cases or the mid‐lateral segments (Figure 3c and Supporting Information: Figure S3C).

### LV phenotypes and the right heart network in PAH

**Figure 4:**
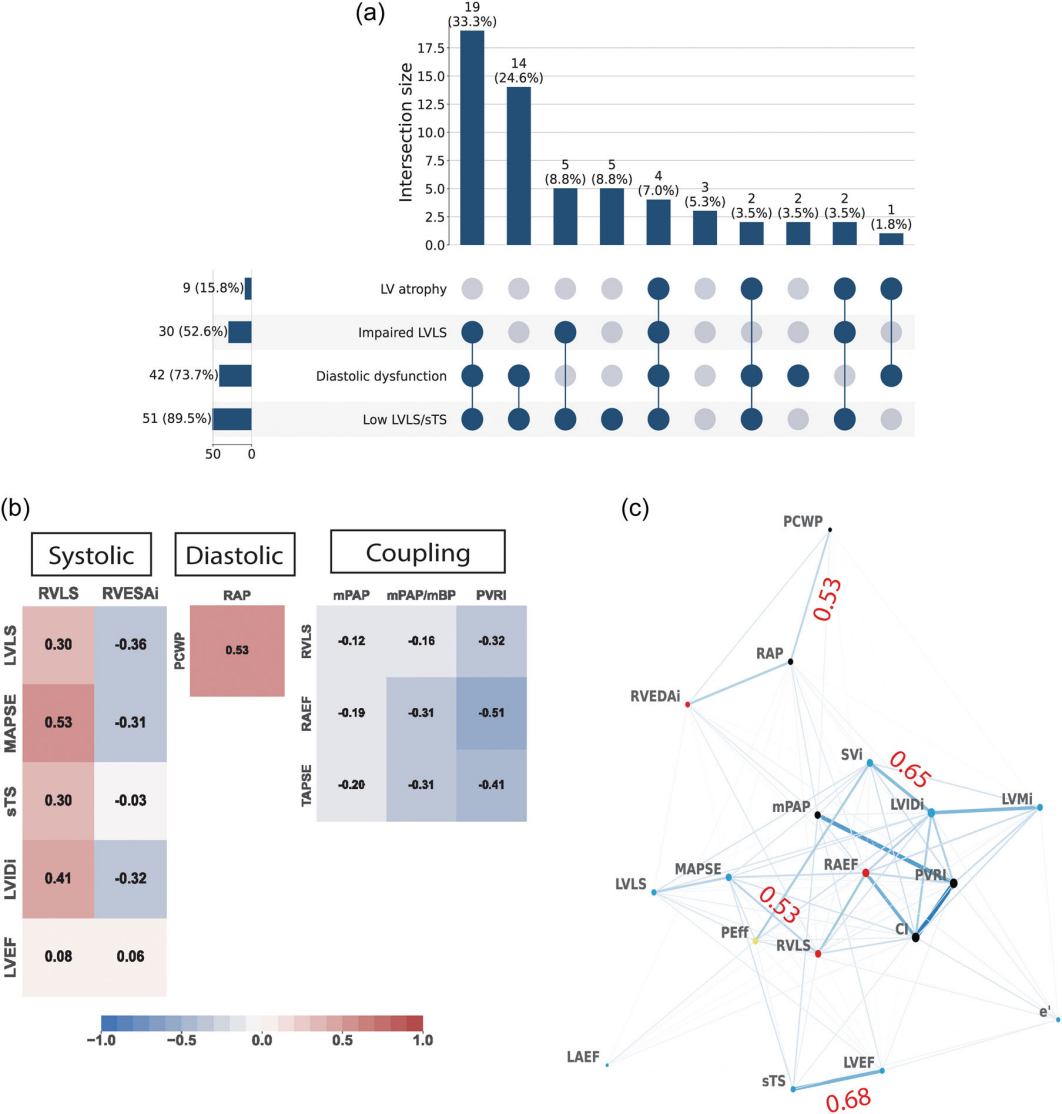
UpSet plot showing different left ventricular (LV) phenotypes in patients with pulmonary arterial hypertension (PAH) and network analysis of the echocardiographic parameters of LV and right ventricular (RV) along with hemodynamic parameters. (a) UpSet plot categorizing the patients based on the presence of LV atrophy, diastolic dysfunction, and either impaired left ventricular longitudinal strain (LVLS) and low LVLS/septal transverse shortening (sTS). The number without parentheses shows the number of patients in each category. (b) Correlation map was created with Spearman's correlation. Correlation coefficient (*r*) is overlaid on the map. (c) Correlation coefficient (*r*) is overlaid with the network (also see Supporting Information: Figure S5). *N* = 57. LVID, LV internal diameter at end‐diastole indexed with height^1.7^; LVMi, LV mass indexed with height^1.7^; PEff, pericardial effusion; RAEF, right atrial emptying fraction; RVEDAi, right ventricular end‐diastolic area indexed with height^1.7^; SVi, stroke volume indexed with height^1.7^.

Upset plots summarize the prevalence of individual features as well the prevalence of their combination. We selected four key features, that is, LVLS, LVLS/sTS ratio, diastolic function, and LV mass index to define ventricular abnormalities. The most common abnormalities were an impaired ratio of LVLS/sTS (89.5%), followed by diastolic dysfunction (73.7%), impaired LVLS (52.6%), and LV atrophy (15.8%) as shown in Figure 4a. The most common combination was abnormal LVLS/sTS and diastolic dysfunction (68.4%). The UpSet plot with MAPSE and MAPSE/sTS instead of LVLS and LVLS/sTS showed that all patients had abnormal MAPSE/sTS (Supporting Information: Figure S4).

The LV abnormalities in PAH were not independent of RV function and hemodynamics. The correlation maps in Figure 4b highlight associations most consistent with diastolic and systolic ventricular interdependence as well as ventriculoarterial coupling. More specifically, there was a moderate association between right atrial pressure and pulmonary capillary wedge pressure (*r* = 0.53, *p* < 0.001), between RVLS and MAPSE (*r* = 0.53, *p* < 0.001) or LVLS (*r* = 0.30, *p* = 0.009), and between PVRI and RVLS (*r* = −0.32, *p* = 0.025). In contrast, among LV parameters, the correlation between sTS and LVLS (*r* = 0.13, *p* = 0.395) or MAPSE (*r* = 0.41, *p* = 0.001), as well as between LVEF and LVLS (*r* = 0.31, *p* = 0.009) or MAPSE (*r* = 0.23, *p* = 0.001) were weaker (Supporting Information: Figure S5). Another important finding was that there was no correlation between LVLS/sTS and LA volume (*r* = 0.05, *p* = 0.966) or PCWP (*r* = −0.01, *p* = 0.305). In addition, pericardial effusion, a known predictor of mortality in PAH, was also found to be inversely related to stroke volume index (*r* = −0.57, *p* < 0.001).

### Longitudinal trajectory in patients with PAH

We selected three groups of representative samples who had NT‐proBNP and echocardiography within a 1–2‐week period in stable clinical conditions. The baseline NT‐proBNP levels were significantly higher in the improved group compared to the worsened group (*p* = 0.036; Supporting Information: Table S2), while there was no significant difference between the other groups.

**Figure 5:**
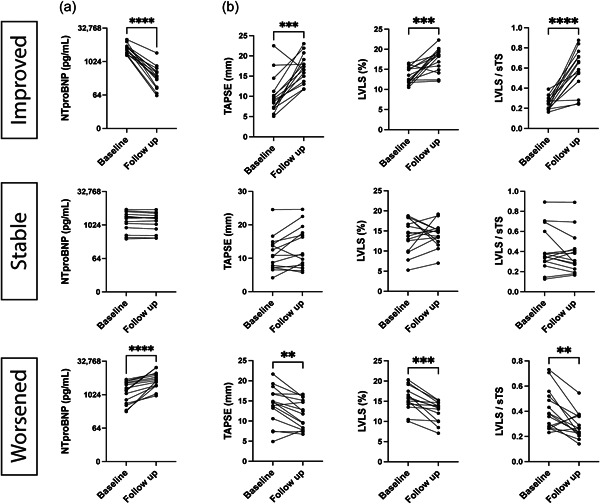
Longitudinal follow‐up of the mechanical shortening indices in pulmonary arterial hypertension (PAH) patients. Patients were categorized into three groups based on their N‐terminal pro B‐type natriuretic peptide (NT‐proBNP) changes in 6–12 months follow‐up. (a) Changes in NT‐proBNP are shown in the logarithmic scale. (b) Changes in tricuspid annular plane systolic excursion (TAPSE), left ventricular longitudinal strain (LVLS), and LVLS/septal transverse shortening (sTS). *N* = 15 for each group. Data were analyzed by Wilcoxon's matched‐pair signed‐rank test. **p < 0.005; ***p < 0.0005; ****p < 0.00005.

When categorizing the patients according to the longitudinal NT‐proBNP group, the improved group showed a significant improvement in TAPSE (*p* = 0.0009), LVLS (*p* = 0.0004), and LVLS/sTS (*p* < 0.0001), whereas the worsened group showed a decrease in all parameters (*p* = 0.0052, *p* = 0.0001, *p* = 0.0020, respectively; Figure 5). In comparison, the stable group showed no change in TAPSE (*p* = 0.068), LVLS (*p* = 0.80), and LVLS/sTS (*p* = 0.33) on paired analysis.

When grouped together, the longitudinal change of NT‐proBNP (logarithmic) was moderately associated with changes of TAPSE (*r* = 0.64, *p* < 0.001), LVLS (*r* = 0.62, *p* < 0.001), and LVLS/sTS (*r* = 0.73, *p* < 0.001) (Supporting Information: Figure S6A). We also saw a moderate correlation between the longitudinal change of TAPSE and LVLS/sTS (*r* = 0.53, *p* < 0.001) (Supporting Information: Figure S6B).

At 5 years, the combined outcome (death or transplantation) occurred in 19 patients (42%). A higher increase in NT‐proBNP over time was related to a higher risk for an outcome (hazard ratio [HR] per one logarithmic SD increase: 2.20, 1.22–2.81, *p* = 0.004) as well as a higher longitudinal increase in TAPSE (HR per 1 − SD increase: 0.56, 0.34–0.93, *p* = 0.030) and LVLS/sTS (HR per unit increase: 0.46, 0.26–0.82, *p* = 0.008).

### LV mechanical shortening indices in a mouse model of RV pressure overload

**Figure 6:**
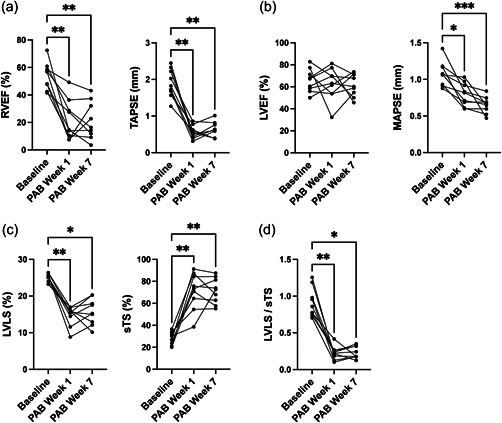
Left ventricular (LV) mechanical shortening indices in pulmonary arterial banding (PAB) mice model assessed by cardiac magnetic resonance imaging (MRI). (a) Right ventricular (RV) dysfunction was noted as a decline in right ventricular ejection fraction (RVEF) and tricuspid annular plane systolic excursion (TAPSE) post‐PAB. (b) LV dysfunction in PAB mice was noted by decreased mitral annular plane systolic excursion (MAPSE) but not by left ventricular ejection fraction (LVEF). (c) LV mechanical shortening in PAB animals was characterized by impaired left ventricular longitudinal strain (LVLS) and increased septal transverse shortening (sTS). (d) LVLS/sTS showed a marked decline early after PAB. The graph shows *N* = 9 animals with longitudinal follow‐up. Data were analyzed by Friedman test with Dunn's multiple comparisons. *p < 0.05; **p < 0.005; ***p < 0.0005.

Last, we explored whether similar findings could be recapitulated in a small animal model of RV pressure overload‐induced RV failure (i.e., PAB). RVEF and TAPSE were significantly decreased at 1‐week post‐PAB and remained impaired until week 7 (Figure 6a). LVEF did not change significantly, while MAPSE and LVLS were both significantly decreased already at 1‐week post‐PAB (Figure 6b,c). On the other hand, sTS was significantly increased (Figure 6c). Accordingly, LVLS/sTS showed a separation between baseline and post‐PAB state (Figure 6d) with a strong correlation with disease severity defined by low RVEF and LV stroke volume (Supporting Information: Figure S7B).

## DISCUSSION

In our study, we showed that the ratio of LVLS/sTS provides novel insights into systolic ventricular interdependence in PAH. The LVLS/sTS ratio emerged as a sensitive marker of LV dysfunction in PAH, reflecting both delayed septal displacement and interdependence in the annular excursion. Our longitudinal analysis further demonstrated the extent of ventricular interdependence in PAH as changes in LVLS/sTS were closely related to changes in TAPSE and NT‐proBNP. Finally, we demonstrated that similar findings of LV mechanics could be recapitulated in a mouse model of RV pressure overload.

Ventricular interdependence plays a central role in PAH. In 1967, Tayler et al.^26^ showed in an explanted canine heart model that LV filling was impaired proportionally with the increase in RV pressure. This was followed by clinical studies showing that patients with PAH have prolonged ventricular relaxation shown as decreased *E*/*A* ratio^27^ and decreased LV isovolumic relaxation time.^28^ Another aspect of LV dysfunction studied in PAH is impairment in LV systolic function.^10^ While LVEF is known to be preserved or increased in PAH until late in its course, abnormal LVLS have been reported in multiple studies using speckle‐tracking echocardiography,^7^, ^10^, ^29^ as well as in studies using feature‐tracking cardiac MRI.^30^, ^31^, ^32^

In our study, we introduce a novel index of mechanical shortening that captures both annular interdependence and late systolic septal displacement. Several mechanistic studies have in fact documented the prolonged contraction of the RV that occurs with pressure overload leading to a late systolic shift.^33^ This is well visualized in the time‐series domain showing the clearly different profile between transverse displacement and LS. Relating LVLS and ejection fraction is not novel in the field of mechanical ventricular analysis. For example, in amyloidosis, the ratio of LVEF/LVLS has been shown to be a more sensitive marker of systolic dysfunction than LVLS.^34^ The justification in amyloidosis or hypertensive heart disease takes advantage of the fact that LVEF usually increases in the presence of LV hypertrophy, while LVLS is impaired. Using mathematical models, Stokke et al.^35^ have shown that LVLS should increase at higher values of LVEF based on the geometrical relationship between circumferential shortening and LS. One pitfall of using ratios is the potential of pseudonormalization, which could occur in our case in late‐stage PAH when both LVLS and sTS are decreased. However, we did not observe pseudonormalization in the three patients with low LVEF in our cohort.

The other important contribution of our study is that we defined abnormalities of the LV from the perspective of LV phenotypes. We focused on three major phenotypes: LV atrophy, LV diastolic dysfunction, and abnormal LV mechanical shortening. The presence of LV atrophy has been reported in PAH,^36^ which has been shown not only by clinical imaging but also using in vitro experiments of human cardiomyocytes isolated from patients with PAH.^37^ We demonstrated to which extent these phenotypes are common in PAH. We found that all patients in our cohort who had a wide range of pulmonary vascular resistance had at least one abnormality, the most common being lower values of LVLS/sTS or MAPSE/sTS. Although diastolic dysfunction was the second common phenotype in our cohort, *E*/*e′* (Table 2) and PCWP (Table 1) were preserved in the majority of PAH cases, which is in line with the previous report by Gurudevan et al.^38^ Alongside the fact that the correlations between LVLS/sTS and LA volume or PCWP were poor, this finding supports the hypothesis proposed by Gurudevan et al. that LV compression by enlarged RV is not the major determinant of abnormal E/A.

Another contribution of our study is that we showed how the longitudinal changes in NT‐proBNP relate not only to TAPSE but also to LVLS and LVLS/sTS. This supports the importance of ventricular interdependence and dynamic coupling in PAH. It supports the important concept that response in PAH should not be viewed in isolation but in the context of consistent ventricular response profiles. Despite our small sample size, changes in NT‐proBNP, TAPSE, and LVLS/sTS all correlated to survival, which further highlights the importance of ventricular interactions in PAH.

Our study has both clinical and translational implications. For clinical implication, our study shows that when analyzing LV systolic function in PAH, the focus should be redirected to LS and TS to describe early abnormalities. We further demonstrate that longitudinal changes in LV function often present parallel changes in RV function; this can be valuable when monitoring patients with PAH and refocuses analysis not on a specific parameter but rather on a consistent response profile. For translational studies, we also showed consistency between LV parameters in a preclinical small animal model and clinical studies in patients with PAH demonstrating the feasibility of studying the LV mechanics as well as possible interventions in the PAB mouse model.

Our study has two major limitations. First, the cohort size was relatively small due to the limited availability of echocardiograms and invasive RHC within a narrow time window. Also, the semiautomatic tracking was suboptimal in some cases due to image quality as we required optimal tracking of all six segments for the analysis of the time‐series events.

In conclusion, we demonstrated that the ratio of LVLS/sTS provides novel insights into ventricular interdependence in PAH. If further developed, future studies can develop automated ways to analyze and be integrated into clinical practice.

## AUTHOR CONTRIBUTIONS

Kenzo Ichimura and Francois Haddad contributed to the study design. Kenzo Ichimura, Everton J. Santana, Nicholas Cauwenberghs, František Sabovčik, Lindsey Chun, Nadia L. C. Francisco acquired and analyzed the data. Kenzo Ichimura, Everton J. Santana, and Francois Haddad conceptualized the study conception, interpreted the data, and participated in drafting the manuscript. Tatiana Kuznetsova, Vitaly O. Kheyfets, Michael Salerno, Roham T. Zamanian, and Everton J. Santana revised it critically for important intellectual content. All authors approved the final version for publication and take responsibility for appropriate portions of the content.

## CONFLICT OF INTEREST STATEMENT

Francois Haddad reports research grants from Actelion Pharmaceuticals, a Janssen Company of Johnson & Johnson focused on computational approaches for the diagnosis and monitoring of pulmonary hypertension. The other authors declare no conflict of interest.

## ETHICS STATEMENT

This study was approved by the Stanford University Institutional Review Board (IRB #14083, #20942) and all patients gave written informed consent.

## Supporting information

Supporting information.

## Data Availability

The data that support the findings of this study are available from the corresponding author upon reasonable request.
