# Supplementary material for: Novel left ventricular mechanical index in pulmonary arterial hypertension
Source: Pulm Circ. 2023 Apr 1;13(2):e12216. doi: 10.1002/pul2.12216 (PMC10103585; doi:10.1002/pul2.12216)
Supplement: Supplementary file 1 — Supporting information. [file PUL2-13-e12216-s001.docx]

**Supplemental Materials**

**Supplemental Methods**

**Regional Analysis of the LV by Feature Tracking**

In order to minimize signal noise and to normalize signals captured in different sample rates, we interpolated the data points obtained from time series curves generated by TomTec semi-automated feature tracking tool by using cubic spline interpolation and by normalizing the time frame to the RR interval. TS was measured based on the transverse displacement (TD) of each segment using the following calculation:

[
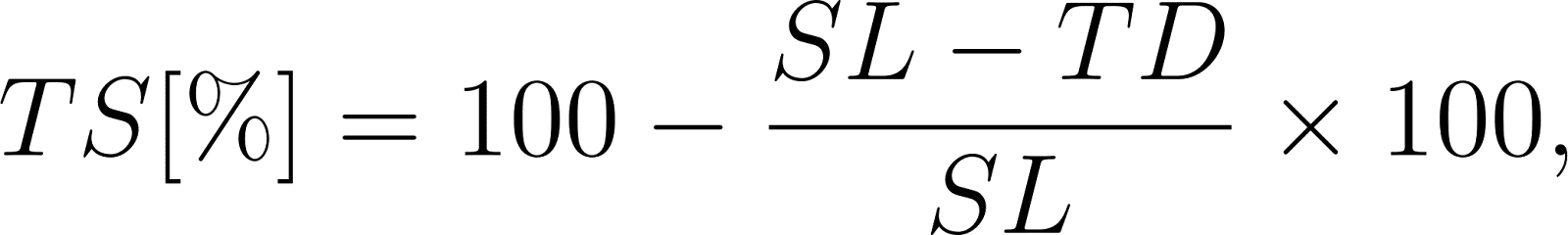
](https://www.codecogs.com/eqnedit.php?latex=TS%20%5B%25%5D%20%3D%20100%20-%20%5Cfrac%7BSL-TD%7D%7BSL%7D%20%5Ctimes%20100%2C#0)

in which SL stands for the mid-LV segment length.

The following parameters from the normalized LS and TS curves were extracted for regional analysis; peak LS and TS of mid-septal and mid-lateral segments (mid-septal LS, mid-septal TS, mid-lateral LS, and mid-lateral TS, respectively); time to peak of LS and TS of each segment.

**Table S1: LV diastolic dysfunction determination criteria**

| **Age** | **E/A (2.5^th^)** | **e’ (2.5^th^)** |
| --- | --- | --- |
| <30 | 1.2 | 12.0 |
| 30-39 | 1.0 | 10.0 |
| 40-49 | 0.9 | 9.9 |
| 50-59 | 0.8 | 7.5 |
| ≥60 | 0.7 | 7.0 |

**Table S2: Comparative echocardiograms of patients in longitudinal cohort at baseline**

|  | Improved (N=15) | Stable (N=15) | Worsened (N=15) |
| --- | --- | --- | --- |
| Age | 50.0 [44.5-58.6] | 48.0 [39.0-61.2] | 54.0 [50.0-59.0] |
| Female sex | 14 (93.3%) | 11 (73.3%) | 13 (86.6%) |
| BMI (kg/m^2^) | 30.7 [25.2-34.1] | 25.1 [23.1-29.6] | 27.8 [25.0-31.1] |
| **PAH etiology** |  |  |  |
| Idiopathic | 4 (26.7%) | 1 (6.6%) | 3 (20.0%) |
| Drugs and toxins | 5 (33.3%) | 5 (33.3%) | 3 (20.0%) |
| Connective tissue disease | 6 (40.0%) | 6 (40.0%) | 8 (53.3%) |
| Congenital heart disease | 0 (0%) | 3 (20.0%) | 1 (6.6%) |
|  |  |  |  |
| 6MWD (m) | 276.9 ± 149.2 | 300.2 ± 145.1 | 340.4 ± 85.9 |
| NYHA class | 3.0 [3.0-3.0] | 3.0 [3.0-3.0] | 3.0 [3.0-3.0] |
| NT-proBNP (pg/mL) | 3514 [2212-4776] | 2279 [776-3076] | 1687 [414-3686] * |
| DLCO (mL) | 69 [42-83] | 63 [50-84] | 64 [37-76] |
|  |  |  |  |
| **Hemodynamics** |  |  |  |
| Systolic blood pressure (mmHg) | 123.5 ± 21.6 | 113.5 ± 11.6 | 115.9 ± 9.61 |
| HR (bpm) | 91.6 ± 17.3 | 80.1 ± 14.9 | 81.8 ± 15.4 |
| RAP (mmHg) | 11.5 ± 6.3 | 11.0 ± 7.5 | 7.8 ± 3.8 |
| mPAP (mmHg) | 55 [45-59] | 57 [46-68] | 51 [36-60] |
| PCWP (mmHg) | 10.4 ± 3.9 | 12.3 ± 7.1 | 8.7 ± 3.1 |
| PVRI (W.U. m^2^) | 26.7 ± 7.6 | 25.6 ± 9.9 | 25.3 ± 14.1 |
| CI (l/min/m^2^) | 1.6 [1.4-2.0] | 1.7 [1.5-1.9] | 1.9 [1.5-2.3] |
| SVRI (W.U./m^2^) | 49.9 ± 14.9 | 51.7 ± 15.0 | 44.1 ±11.0 |
|  |  |  |  |
| LVID (mm) | 38.8 ± 7.9 | 38.5 ± 6.5 | 37.7 ± 5.5 |
| LVPWT (mm) | 9.0 ± 0.8 | 8.5 ± 1.5 | 9.9 ± 2.2 |
| LVEF (%) | 63.9 ± 6.4 | 61.9 ± 10.3 | 64.5 ± 11.2 |
| LVSV (mL) | 36.4 [30.0-47.4] | 36.8 [30.4-41.7] | 48.4 [29.5-56.3] |
| LVLS (%) | 13.4 ± 1.99 | 13.9 ± 4.18 | 15.7 ± 2.95 |
| MAPSE (mm) | 7.3 ± 2.30 | 7.6 ± 2.52 | 8.4 ± 2.70 |
|  |  |  |  |
| RVEDA (mm^2^) | 34.6 ± 10.4 | 41.2 ± 20.3 | 35.7 ± 9.9 |
| RVFAC (%) | 22.6 ± 4.6 | 24.4 ± 8.8 | 24.5 ± 6.1 |
| RVLS (%) | 13.5 [12.2-14.7] | 14.0 [10.0-16.3] | 14.2 [10.8-18.4] |

 *: p<0.05 vs Improved

**
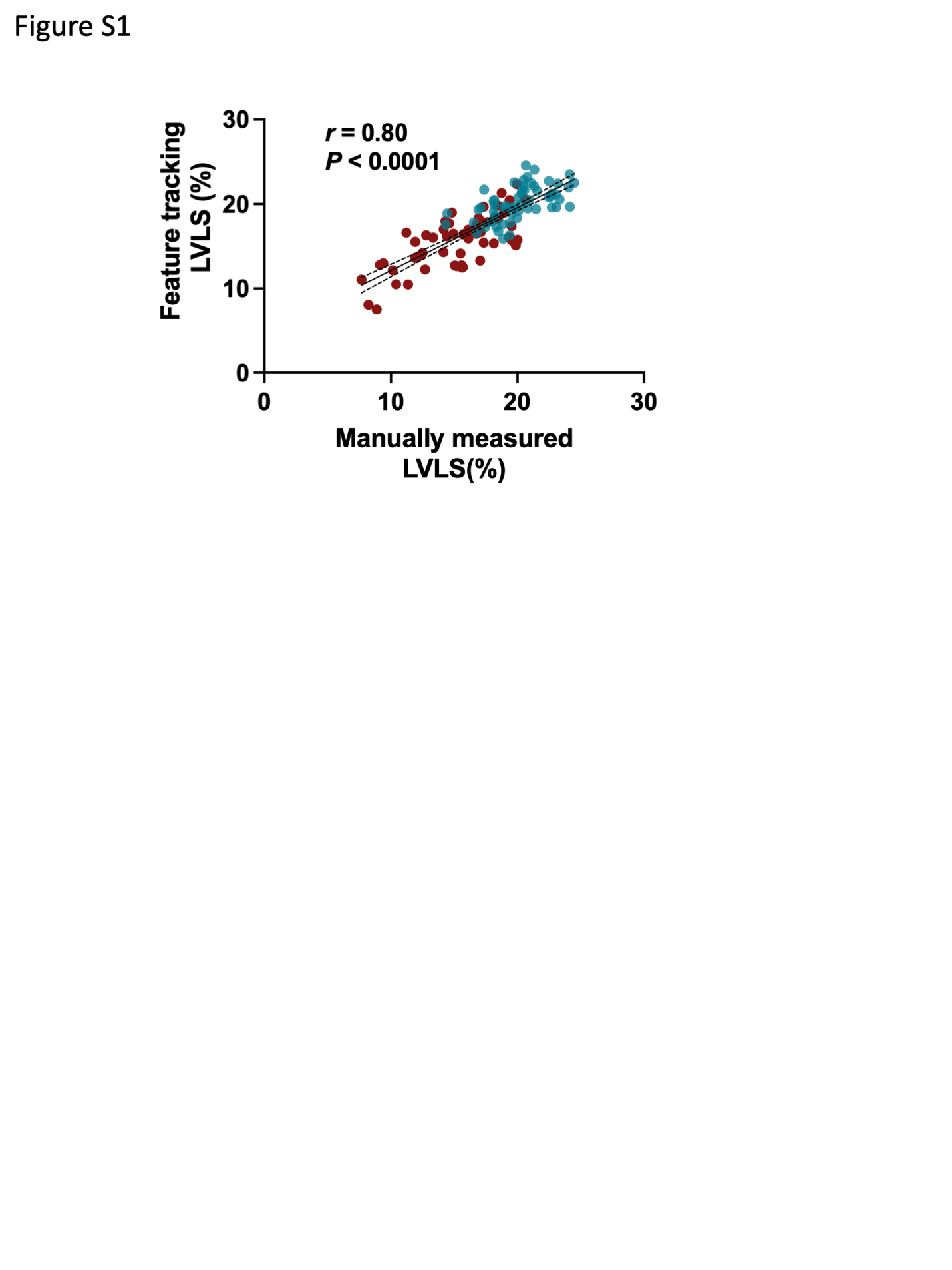
**

**Figure S1: Correlation of LVLS measured manually by Lagrangian method and by semi-automated feature tracking**

Simple linear regression showing the correlation between LVLS measured manually by Lagrangian method and by semi-automated feature tracking of TomTec. Cyan dots represent healthy control cases (N=50) and dark-red dots represent patients with PAH (N= 48).

**Figure S2: Correlation of different LV shortening indices and hemodynamics in patients with PAH**

Simple linear regression showing the correlation between three LV shortening indices and hemodynamics. N= 57.

**Figure S3: Regional displacement, strain, and time dispersion analysis of the LV mid-lateral wall by automated feature tracking**

LV shortening was assessed in both amplitude (A) and time (B) domain. **A:** Minor decrease in LS was noted in the mid-lateral wall of PAH patients, whereas TS was comparable between groups. **B:** Although the time to peak was delayed both in LS and TS in PAH patients compared to control, time dispersion between LS and TS was not observed. Data were analyzed by unpaired two-tailed Mann-Whitney *U*-test. *****:** *P* <0.0005, ****: *P* <0.00005. **C:** Representative curves of LS and TS of mid-lateral segments.

**Figure S4: UpSet plot with MAPSE and MAPSE/sTS showing different LV phenotypes in patients with PAH**

UpSet plot categorizing the patients based on the presence of LV atrophy, diastolic dysfunction, and impaired MAPSE and low MASPE/sTS. The number without parentheses shows the N of patients in each category.  LV atrophy was defined as 2.5^th^ percentile of the normal value. LVLS below 16% in absolute value was defined as abnormal based on the guideline. Thresholds of low MAPSE, and MAPSE/sTS were defined as 2.5^th^ percentile of the control cases. Diastolic dysfunction was determined if E/A or e’ was abnormal (Supplemental Table). N=57.

**Figure S5: Correlation map of the parameters used for network analysis**

Correlation map created with Spearman’s correlation. Correlation coefficient (*r*) is overlaid in the map. N=57. LAVi: left atrial volume indexed with height^1.7^, LVIDi: LV internal diameter at end diastole indexed with height^1.7^, LVMi: LV mass indexed with height^1.7^, LVPER: left ventricular peak ejection rate, LVPFR: left ventricular peak filling rate, PEff: pericardial effusion, RAEF: right atrial emptying fraction, RVEDAi: right ventricular end diastolic area indexed with height^1.7^, SVi: stroke volume indexed with height^1.7^.


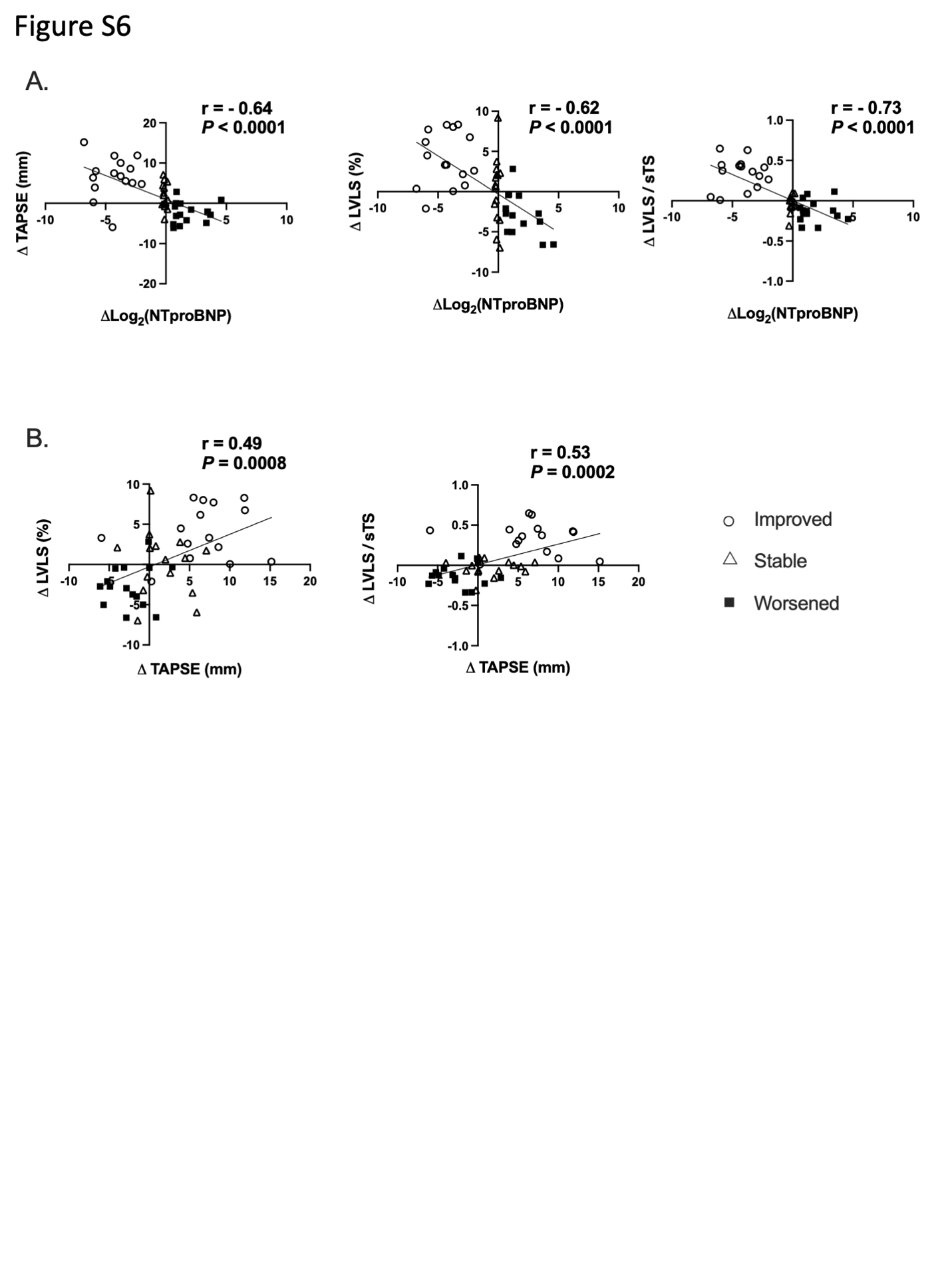


**Figure S6: Correlation between the longitudinal changes of NT-proBNP, TAPSE, LVLS, and LVLS/sTS**

**A.** Simple linear regression showing the correlation between the longitudinal changes of NT-proBNP (logarithmic changes) and TAPSE, LVLS, and LVLS/sTS. N= 45. **B.** Simple linear regression showing the correlation between the longitudinal changes of TAPSE, LVLS, and LVLS/sTS. N=45.

**Figure S7: LV mechanical shortening indices and correlation map of LV and RV parameters in PAB mice model assessed by cardiac MRI**

**A:** LVLS/LVEF and MAPSE/sTS showed a marked decline early after PAB. The graph shows N=9 animals with longitudinal follow-up. Data were analyzed by Friedman test with Dunn’s multiple comparisons. *: *P* <0.05, **: *P* <0.005, ***: *P* <0.0005. **B:** Correlation map created with Spearman’s correlation. Correlation coefficient (*r*) is overlaid in the map. N=27 (three time points/animal).
